# Value of preclinical systematic reviews and meta-analyses in pediatric research

**DOI:** 10.1038/s41390-024-03197-1

**Published:** 2024-04-13

**Authors:** Olga Romantsik, Matthias Bank, Julia M. L. Menon, Atul Malhotra, Matteo Bruschettini

**Affiliations:** 1 Department of Clinical Sciences Lund, Division of Pediatrics, Lund University, Skåne University Hospital, Lund, 21185 Sweden; 2 Library and ICT, Faculty of Medicine, Lund University, Lund, Sweden; 3 Preclinicaltrials.eu, Netherlands Heart Institute, Utrecht, The Netherlands; 4 Department of Pediatrics, Monash University, Melbourne, Australia; 5 Monash Newborn, Monash Children’s Hospital, Melbourne, Australia; 6 The Ritchie Centre, Hudson Institute of Medical Research, Melbourne, Australia

## Abstract

**Abstract:**

Similar to systematic reviews (SRs) in clinical fields, preclinical SRs address a specific research area, furnishing information on current knowledge, possible gaps, and potential methodological flaws of study design, conduct, and report. One of the main goals of preclinical SRs is to identify aspiring treatment strategies and evaluate if currently available data is solid enough to translate to clinical trials or highlight the gaps, thus justifying the need for new studies. It is imperative to rigorously follow the methodological standards that are widely available. These include registration of the protocol and adherence to guidelines for assessing the risk of bias, study quality, and certainty of evidence. A special consideration should be made for pediatric SRs, clinical and preclinical, due to the unique characteristics of this age group. These include rationale for intervention and comparison of primary and secondary outcomes. Outcomes measured should acknowledge age-related physiological changes and maturational processes of different organ systems. It is crucial to choose the age of the animals appropriately and its possible correspondence for specific pediatric age groups. The findings of well-conducted SRs of preclinical studies have the potential to provide a reliable evidence synthesis to guide the design of future preclinical and clinical studies.

**Impact:**

- This narrative review highlights the importance of rigorous design, conduct and reporting of preclinical primary studies and systematic reviews.
- A special consideration should be made for pediatric systematic reviews of preclinical studies, due to the unique characteristics of this age group.

## Introduction

**Fig. 1:**
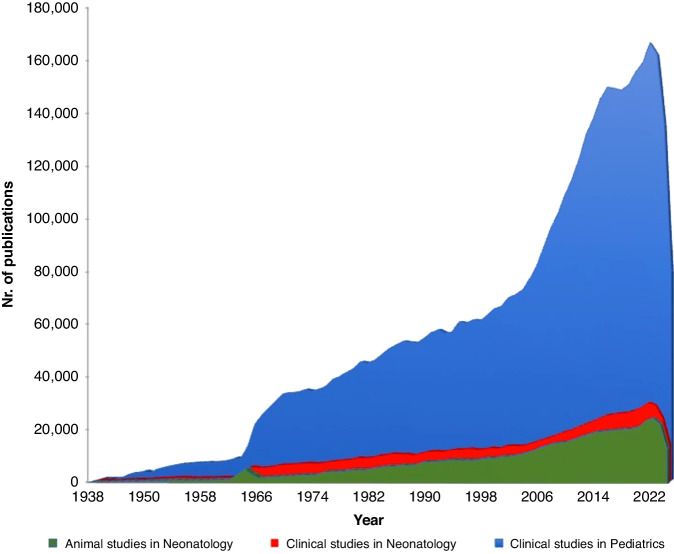
Number of studies published in pediatrics and neonatology over the years (range 1938–2022). The search of PubMed was performed on October 17, 2023.

It is extremely challenging to keep up to date with medical literature due to the high publication rate and data overload. More than 4.6 million papers are available on PubMed for different medical conditions in children (birth - 18 years). The rate of publications is increasing exponentially, starting with low numbers of publications for many decades and reaching more than 130000 publications in 2022 (Fig. 1). Electronic scoping searches of PubMed were performed on October 17, 2023 (Supplementary material). This trend presents in many medical branches^1,2^ implies a great challenge for healthcare professionals to keep updated with the progress in their specific fields. Consequently, one relies on reviews (both narrative and systematic) to get an overview of the specific topic.

In this paper, we focus on preclinical systematic reviews (SRs) which pertain to pediatric medicine. We start with an overview of reviews including clinical SRs, unique preclinical SRs features, and those that apply especially to pediatric medicine. Emphasis on the quality of SRs is reiterated.

SRs and meta-analyses systematically scrutinize available literature on the topic and evaluate the study limitations of the reported data, following a detailed protocol. SRs are based on robust methodology, starting with a well-defined review question, which steers the criteria for a comprehensive literature search and the precise inclusion and exclusion criteria.^3^ Thus, the reader gets the opportunity to reproduce the search and understand the selection of the papers. In addition, two people independently conduct the screening, extract relevant outcome data, and evaluate the risk of bias and certainty of the evidence, thus minimizing the risk of introducing an error in the process. The meta-analysis, where possible, allows to calculate the effect size for each outcome. Furthermore, the SR points out the possible presence of publication bias. SRs even report the characteristics of ongoing studies on the specific topic. By doing so, it is easier for readers to follow up on the latest developments in the field. With this premise in mind, SRs and meta-analyses are valuable tools, providing a systematic assessment of the specific questions, highlighting the knowledge gaps, and addressing independently the quality of published science, thereby raising awareness of research waste caused by studies of mediocre quality.

Despite this exponential growth of SRs in clinical medicine, they are less common in preclinical medicine (in vitro studies, animal studies, and ex-vivo studies).^4^ The first SR of animal studies was published by Omarini et al. in 1992.^5^ Their SR regarded the placental perfusion in seven different animal species either in situ or in vitro. Freedman et al. published the first meta-analysis of animal studies on the effects of dietary fat consumption on mammary tumor development in 1994.^6^ Since then, approximately 3000 SRs in animal studies have been published, and approximately one-third of those included a meta-analysis.^4^ Hunniford et al. reported in their epidemiological study conducted in 2015–2018 that approximately 54% of all preclinical SRs focused on pharmacological interventions and 46% on non-pharmacological interventions, mainly cell therapies, and surgery.^7^

### Is there a need for preclinical SRs?

Similar to SRs in the clinical field, preclinical SRs address a very specific research area and describe the current knowledge, possible gaps, and flaws of the study design, conduct, and reporting of each included study.^8,9^ By analyzing available data, SRs may prevent the duplication of experiments and thereby reduce research waste and unethical use of animals. Since launching the methodology for preclinical SRs, Radboud University in the Netherlands could reduce the use of research animals by 35% at their institution, and by 15% in the whole country.^10,11^ Additionally, they raised awareness of possible methodological flaws and biases, ideally resulting in improved study design, conduct, and report. Menon et al. demonstrated in their mixed case study that the conduct of preclinical SRs changed the mentality of the surveyed scientists on the quality of animal research, resulting in higher quality and transparency of the following work of the same preclinical researchers. It led to a desire to diffuse this knowledge within their research teams and advocate for the broader education of the scientists.^2^

Given the heterogeneity of preclinical research and multiple animal species used to model different health-related conditions, SRs may be extremely valuable in choosing the most appropriate animal model^12,13^ and outcome measures. SRs anticipate the information if the obtained evidence is sufficient to move the research question into the clinic or illuminate the gaps thereof justifying the need for new studies.^10,14–16^ One could argue that preclinical SRs may act as the bridge between the preclinical and clinical scientific world.

### Quality assessment in clinical SRs

SRs are often valuable evidence sources for clinical guidelines, drug regulation processes, and decision-making tasks for physicians and policymakers, which require high quality.^9,17^ This is why SRs must follow rigorous and detailed guidelines for the summarized evidence to be reproducible and trustworthy. In the clinical field of healthcare, two international organizations Cochrane (formerly Cochrane Collaboration; https://www.cochrane.org) and JBI (formerly Joanna Briggs Institute, https://jbi.global/) provide the criteria and methodological standards for assessment of current evidence, periodically updating their methods based on the reflection of the new information and ever-changing needs. Importantly, they use the Grading of Recommendations Assessment, Development, and Evaluation (GRADE) system to assess the certainty of the presented evidence.^18^ The GRADE working group, which consists of various healthcare professionals, methodologists, guidelines developers, healthcare researchers, and economists, developed and implemented “a common, transparent and sensible approach to grade the quality of evidence and strengths of recommendations in healthcare”.^18^ Well-defined protocols and checklists have to be followed, involving a multi-step, peer-review process.^19,20^

Despite being the largest database for SRs in clinical medicine, the Cochrane Database of Systematic Reviews accounts only for 7% of published SRs.^21^ To date, the World Health Organization demands Cochrane standards to summarize the evidence for the development of their clinical practice guidelines.^22^ It has been reported that the quality of Cochrane reviews is superior to non-Cochrane reviews.^9,23,24^ For example, Kolaski et al. assessed the quality of SRs of interventions for children with cerebral palsy using the Measurement Tool to Assess Systematic Reviews-2 (AMSTAR-2).^24,25^ Eighty-three SRs were included in their analysis, four of these were Cochrane reviews. The only reviews that were approved by the AMSTAR-2 tool^25^ were published within Cochrane, the remaining SRs were deficient for critical and non-critical domains of AMSTAR-2 evaluation. This implies that recommendations on the treatment of children with cerebral palsy are based on critically low quality of evidence.^24^ One of the critical items in the AMSTAR-2 quality assessment tool is the publication of the SR protocol before conducting SR.^25^ Protocol registration increases the transparency and quality of the research and diminishes the risk of duplication, and potential misconduct.^26^ It thus appears to result in higher quality methodology.^27–29^ Protocol registration is mandatory for Cochrane reviews^3^ but rarely for non-Cochrane SRs, depending on journal requirements. To overcome this problem the Prospective Register of Systematic Reviews (PROSPERO, https://www.crd.york.ac.uk/prospero/) was launched in 2011.^29,30^ It is a free open database for the registration of protocols for SRs associated with health care. Differently to clinical trials on humans where the registration of protocol is obligatory,^31^ there is no such requirement for SRs. Indeed, according to a recent study by van der Braak et al., only 38% of SRs on interventions published between January 2020 and January 2021 had a preregistered/ published protocol.^29^ This percentage is increasing compared to 5.6% in 2013 (no protocols for SRs were found before 2013), and 31.6% in 2018.^32^

A tool to assess the risk of bias within SRs is the Risk of Bias in Systematic Reviews (ROBIS).^33^ Differently from the AMSTAR-2 that is applied for the intervention SRs,^34^ ROBIS may be applied for intervention, diagnostic, etiology, and prognostic SRs.^33^ The two tools are related and have several overlapping domains, however, they are not interchangeable. Both tools pinpoint the methodological quality (the prevention of systematic errors by study design, conduction, analysis, interpretation, and publication) and risk of bias (whether the results of the study are affected by the drawbacks in design, conduction, and analysis).^35^ Both AMSTAR-2 and ROBIS demonstrated good inter-rater reliability,^24,25,33,35,36^ being not superior to each other.^36,37^ Indeed, in the overview of SRs on complementary and alternative medicine therapies for infantile colic, inter-rater reliability was 0.6 for AMSTAR-2 and 0.63 for ROBIS.^36^ It is pivotal though to train the authors for AMSTAR-2 and ROBIS to understand the differences between these two methods, and to make the conscious choice of which one to use to follow methodological rigor.

While AMSTAR-2 and ROBIS are crucial tools to assess SR conduct, the Preferred Reporting Items for Systematic Reviews and Meta-Analyses (PRISMA) is the guideline for the reporting of SR.^38,39^ The updated version of the PRISMA guideline includes 27 items within seven sections (title, abstract, introduction, methods, results, discussion, and other information).^38^ The acknowledgment of PRISMA guidelines is beneficial already in the planning phase of a SR to ensure that all required items are covered and the appropriate methodological choices are made.^9,38^ Following the PRISMA guidelines allows the authors to generate a complete and transparent reporting of their SR.

Importantly, the PRISMA checklist determines how completely each of the seven sections of SR is reported, but does not ascertain the quality of conduct and performance. Likewise, AMSTAR-2 and ROBIS are tools to assess the conduct of SR but they do not replace the methodological guidance. It has been shown that adherence to the PRISMA checklist does not guarantee achieving AMSTAR-2 and/or ROBIS standards.^40–42^ In a quality assessment study on the timing of complementary feeding for early childhood allergy prevention, it has been demonstrated that only two SRs out of 12 fulfilled all PRISMA 2009 checklist items.^39^ However, both these SRs were assessed to have low and critical low quality assessed by the AMSTAR-2 tool; one of them had a low risk of bias, and the other one high risk of bias assessed by the ROBIS tool.^42^ Therefore, the implementation of AMSTAR-2 and ROBIS for the evaluation of SR conduct and PRISMA for the comprehensiveness of reporting is recommended (Table 1).

**Table 1:** Available tools for clinical and preclinical SRs at planning, conduct and report stage.

Parameter		Clinical SRs	Preclinical SRs
Aim of SR		To implement the intervention into the clinical practice. To help the decision-making and guidelines creation.	“Where we are now in identifying potential treatment strategies/ management?” “Is the currently available data sufficient to proceed to clinical trial?”
Planning of SR	Acknowledgement of reporting requirements	PRISMA (Preferred Reporting Items for Systematic reviews and Meta-Analyses; http://www.prisma-statement.org/)	PRISMA (Preferred Reporting Items for Systematic reviews and Meta-Analyses; http://www.prisma-statement.org/)
	Registration of protocol	Only for SRs: 1. Cochrane database (https://www.cochrane.org) 2. JBI (formerly Joanna Briggs Institute, https://jbi.global/) 3. PROSPERO (Prospective Register of Systematic Reviews; https://www.crd.york.ac.uk/prospero/) 4. Research Registry (https://www.researchregistry.com/register-now/register-your-systematic-review) 5. INPLASY (International Platform of Registered Systematic Review and Meta-analysis Protocols; https://inplasy.com/) All study designs: 1. OSF Registries (https://www.cos.io/initiatives/prereg) 2. protocols.io (https://www.protocols.io/)	Only for SRs: 1. PROSPERO (Prospective Register of Systematic Reviews; https://www.crd.york.ac.uk/prospero/) 2. Research Registry (https://www.researchregistry.com/register-now/register-your-systematic-review) 3. INPLASY (International Platform of Registered Systematic Review and Meta-analysis Protocols; https://inplasy.com/) All study designs: 1. OSF Registries (https://www.cos.io/initiatives/prereg) 2. protocols.io (https://www.protocols.io/) For veterinary SRs: VetSRev (https://vetsrev.nottingham.ac.uk/)
Conduct of SR	Assessment of certainty of evidence	GRADE (Grading of Recommendations Assessment, Development, and Evaluation)	GRADE (Grading of Recommendations Assessment, Development, and Evaluation)
	Quality assessment tool of primary studies	AMSTAR-2 (Measurement Tool to Assess Systematic Reviews-2)	CAMARADES (The Collaborative Approach to Meta-Analysis and Review of Animal Data from Experimental Studies)
	Risk of bias assessment tool	ROBIS (Risk of Bias in Systematic Reviews) RoB2 (Cochrane risk of bias assessment tool)	SYRCLE (Systematic Review Center for Laboratory Animal Experimentation)
Report of SR	Reporting guidelines	PRISMA (Preferred Reporting Items for Systematic reviews and Meta-Analyses; http://www.prisma-statement.org/)	PRISMA (Preferred Reporting Items for Systematic reviews and Meta-Analyses; http://www.prisma-statement.org/)

### Preclinical and clinical SRs: similarities and differences

Preclinical studies aim to understand the pathophysiological processes of the diseases, explore and discover potential treatment strategies, and test the safety and efficacy of new drugs before the initiation of clinical trials.^14,43^ However, the attention to the methodological quality of primary animal studies is still unsatisfactory. Thus, preclinical SRs often have their focus on possible areas in improvement of study design, conduct, and report.

Within pediatrics, the impact of the findings of the SRs on antenatal steroids provides an excellent example. High certainty evidence shows that the administration of antenatal steroids in case of risk of preterm delivery reduces neonatal mortality.^44^ It is useful to look at similarities and discrepancies between the findings of preclinical^45^ and clinical^46^ SRs of antenatal steroids on long-term outcomes. The preclinical SR on antenatal steroids included 64 studies performed mainly in rodents.^45^ The number of primary studies included in this preclinical SR^45^ is twice as high as in clinical SR on antenatal steroids.^46^ However, it is not possible to calculate the total number of animals in this preclinical SR, due to unclear reporting of the sample size in the primary studies (personal communication with Dr. van der Merwe). This is not the case for the clinical SR where the total number of children is reported (1.25 million).^46^ The authors of the two SRs could not perform the subgroup analysis based on sex due to a lack of data in the primary studies.^45,46^ The mortality rate was also underreported in the primary studies included in the preclinical SR (personal communication with Dr. van der Merwe). The lack of information on sex, mortality rate, and how many animals were used at the entry in the primary study raises several ethical questions regarding the completeness of the reporting. The outcomes were measured on term-born animals: animals had mature organ systems and physiology, leading to a further relevant question as a translation of the data into the clinical field. In the clinical study setting betamethasone was the most used antenatal steroid (in 77% of the included studies),^46^ whereas in animal studies dexamethasone was used in 81% of the included studies.^45^ Moreover, only 28% of the animal studies used clinically equivalent doses of steroids.^45^ Two-thirds of studies in animals used multiple courses of steroids^45^ while in clinical studies 1/3 of included studies reported a single dose of antenatal steroids.^46^ Such divergence in the different steroids (betamethasone or dexamethasone), dosage, and administration regimen used between preclinical and clinical studies is problematic. Of note, the authors of the preclinical SR did not perform a meta-analysis of outcome data due to differences in outcome definition, animal model, the dosage of steroids, single/ multiple courses, age of the animal at assessment, and methods of outcome measurement.^45^

#### Quality assurance of preclinical primary studies

To improve the reporting of primary preclinical studies the ARRIVE (Animal Research: Reporting of In Vivo Experiments) guidelines were developed in 2010. The purpose of the ARRIVE guidelines is to increase the quality, reporting, transparency, and reproducibility of primary animal studies.^47^ Endorsement of these guidelines was applied by several journals. Yet, no marked progress was noted by the ARRIVE working group in 2020: randomization was reported by 30–40% of publications, blinding only by 20% of publications, sample size calculation, and basic animal characteristics below 10% of publications.^47^ The authors of the guidelines address two possible reasons for the limited adherence to the guidelines: scarce awareness of the weight of incomplete reporting, and to which extent the journal staff is committed to fulfilling the guidelines.^47^ To defeat the issue of compliance with the ARRIVE guidelines, the ARRIVE working group revised, updated, and reorganized the first version introducing a more user-friendly adaptation of ARRIVE 2.0 guidelines.^47^ They consist of two sets: the “ARRIVE Essential 10” and the “ARRIVE Recommended Set”. The former provides the fundamental requirements for the reliability of the manuscript: study design, sample size, measures to reduce subjective bias, outcome measures, statistical methods, animals, experimental procedures, and results.^47^ The “ARRIVE Recommended Set” invites to provide detailed information on animal husbandry and care, protocol registration, ethical disclosure, and declaration of interests.^47^

One of the possible solutions to these problems is protocol registration, or preregistration.^47–49^ Although widely accepted and used in clinical trials, it is still extremely uncommon in preclinical research. ARRIVE 2.0 guidelines strongly recommend the registration of protocol.^47^ Preregistration of protocol results in reporting on detailed study design, randomization, blinding, primary outcome measure, and planned analysis, which reduces risks of questionable research practices like HARKing (Hypothesizing After the Results are Known^50^ or cherry picking (report of advantageous results with occulting the unfavorable results).^51^ Registration of the primary studies’ protocols is a simple and free procedure, which might be performed in registers such as https://preclinicaltrials.eu/ or https://www.animalstudyregistry.org/.

The consultation with ARRIVE 2.0 guidelines at the protocol stage of the study enhances the chances of higher quality and addresses the potential biases. If the primary outcomes are accurately pre-specified in a-priori published protocol, the obtained data, independently whether it is positive, negative, or neutral, is more reliable.^15,16,47–49,52,53^ Moreover, it can minimize the risk of outcome switching based on results, thus the research remains hypothesis-driven and not result-driven.^49^

Additionally, PREPARE (Planning Research and Experimental Procedures on Animals: Recommendations for Excellence) guidelines, available at https://norecopa.no/prepare, may be used for individual animal studies.^54^ This guideline consists of the following parts: formulation of the study, dialog between scientists and the animal facility, and quality control of the various components of the study.^54^

Recognizing the problem within preclinical research, Nature Publishing Group changed the editorial policy creating a 10-item checklist for manuscript revision that addresses whether certain measurements were applied to assure randomization, blinding, sample size calculation, data analysis, and publication bias, in May 2013.^55^ The follow-up study revealed an improvement in reporting risk of bias by 16.4% in Nature group journals compared to the other types of journals (no change detected).^55^ This indicates that change in acceptance to follow higher study conduct and report standards may be, although slow, possible.

#### Quality assessment in preclinical SRs

The recognition of the above-mentioned problems led to the development of detailed guidance via a free-of-charge online platform (The Systematic Review & Meta-analysis Facility (SyRF), https://syrf.org.uk/) on how to perform a high-quality SRs in animals.^15,48,56–61^ The Collaborative Approach to Meta-Analysis and Review of Animal Data from Experimental Studies (CAMARADES) and Systematic Review Center for Laboratory Animal Experimentation (SYRCLE) have been probably the largest groups, providing methodological assistance (both in means of tools, educational courses, and practical assistance if needed) for the evaluation of animal studies using systematic review approaches.^48,53^ Despite the recent integration of the SYRCLE group into CAMARADES it is still possible to use the SYRCLE tool to assess risk of bias. CAMARADES approaches quality score checklists. It seems that there might be a poor understanding of the differences between these two tools.^1^ The CAMARADES checklist evaluates the reporting by answering pre-specified questions (yes/no, maximum 10) regarding the appropriateness of the animal model, randomization, blinding, sample size calculation, temperature control, compliance with regulatory committees, and statements of conflict of interest.^53^ The SYRCLE’s risk of bias tool uses reporting to look into the risk of bias. It is based on the Cochrane risk of bias assessment tool (a translation from clinical to preclinical SRs research) and it contains 10 bias items (selection, performance, detection, attrition, reporting, and other biases) with possible answers low/high/unclear.^48^ The overview of these two tools is provided in Table 2. Despite the availability of quality and risk of bias assessment tools for the past 20 years, approximately only 45% of SRs in animals have some kind of quality assessment, and around 17% of SRs include both quality assessment and meta-analysis.^4^ The trend of quality assessment in animal studies appears to be promising since the first animal SR publication in 1992,^5^ increasing to 36% in 2010 and 45% in 2019.^4^ Russell et al. reported that only 5% of the SRs published the quality assessment based on the CAMARADES checklist and risk of bias based on SYRCLE.^1^

**Table 2:** CAMARADES quality assessment checklist and SYRCLE risk of bias items.

CAMARADES (MacLeod 2004) Yes/No	SYRCLE (Hooijmans 2014) Yes/No/Unclear
1. Regulatory compliance statement	1. Sequence generation (selection bias)
2. Sample size calculation	2. Baseline characteristics (selection bias)
3. Statement of temperature control	3. Allocation concealment (selection bias)
4. Co-morbid animals	4. Random housing (performance bias)
5. Confirmation method of the model (Originally: “Use of anesthetic without significant intrinsic neuroprotective activity”)	5. Blinding (performance bias)
6. Randomization	6. Random outcome assessment (detection bias)
7. Blinded application of treatment (Originally: “Blinded application of ischemia”)	7. Blinding (detection bias)
8. Blinded assessment of outcomes	8. Incomplete outcome data (attrition bias)
9. Statement of conflict of interest	9. Selective outcome reporting (reporting bias)
10. Peer-reviewed journal	10. Other sources of biases (other, e.g. contamination/pooling drugs, unit of analysis errors, replacement of drop-outs from the original population, design-specific risk of bias, funding)

CAMRADES checklist reported in this table has 2 modifications compared to the original publication: items 5 and 7.

As a result of the adoption and use of SRs in the preclinical field, several common issues became obvious. Lack of randomization and blinding in preclinical studies results in an overestimation of the detected size effect, leading to erroneous and misleading interpretations.^8,53,62–64^ Sample size calculation is scarcely reported and the majority of animal studies on intervention have very few animals per group (e.g. 6–8) resulting in poor statistical power.^1,8,52,56^ On top of that, small studies may result in a greater effect compared to large studies (both in preclinical and clinical settings) due to the heterogeneity.^65–67^ They are more subjected to selection, attrition, and publication biases, resulting in false-positive intervention effects (both in clinical and preclinical research).^24,65–67^ Publication bias, e.g. the studies with positive results are most likely to be published, may lead to overestimation of the intervention effect and potentially duplication of the research due to missing reporting of negative results.^49,68,69^ Mueller et al. reported that just half of the animal SRs assessed publication bias.^69^

Likewise for the primary preclinical studies the registration of protocol for SR is crucial. Indeed, it is one of the domains in the SYRCLE tool on selective outcome reporting.^48^

There are different databases available for animal SR protocol registration: PROSPERO (https://www.crd.york.ac.uk/prospero/), Research Registry (Research Registry - Registry of Systematic Reviews/Meta-Analyses, https://www.researchregistry.com/register-now/register-your-systematic-review), INPLASY (International Platform of Registered Systematic Review and Meta-analysis Protocols, https://inplasy.com/). PROSPERO is free of charge, accepts only SRs with a clear benefit for human health, and allows version tracking. Research Registry and INPLASY are available by payment and both accept SRs, INPLASY accepts additional scoping reviews. All three databases provide a unique identifying number and the data submission section is possible only for the Research Registry.^70^ Two other registers accept all study designs: OSF Registries (OSF preregistration, https://www.cos.io/initiatives/prereg) and protocols.io (https://www.protocols.io/). Both are free of charge and provide version tracking and a DOI.^70^ The result submission is not possible, but OSF Registers provide the link to the OSF projects where the data can be presented.^70^ PROSPERO seems to be the most used with more than 100000 SR protocols registered.^70^ For veterinary SRs specifically, another dedicated register is available, VetSRev (https://vetsrev.nottingham.ac.uk/).

A database for animal SRs was developed by Langendam et al. in 2021, and it is freely available online (https://data.mendeley.com).^4^ The purposes of this database are: “(1) avoid duplication of effort and, thus, reduce research waste, (2) facilitate researchers in easily identifying all systematic reviews on a specific topic, (3) aid in the creation of evidence maps, (4) serve as a resource for further analysis to advance the methodology in evidence synthesis of animal-based research”.^4^ The database contains all SRs in animals since the first publication in 1992. Another question that may be addressed with the help of this database is a translation of animal studies in humans.^4^

**Fig. 2:**
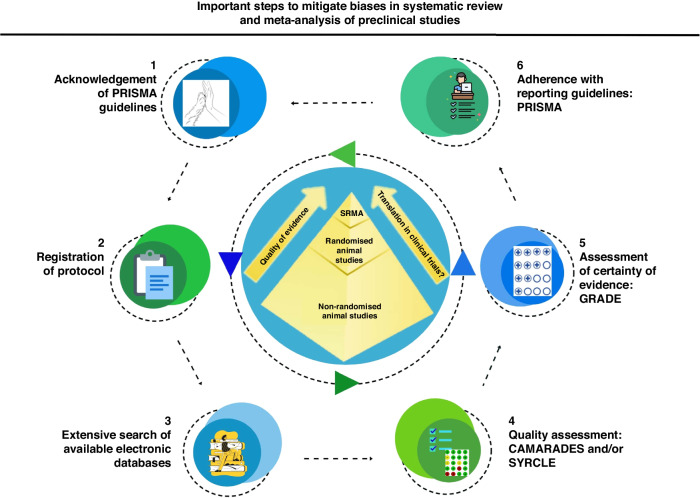
Infographic: ”Important steps to mitigate biases in systematic review and meta-analysis of preclinical studies”. To note: this figure gives some relevant steps to mitigate bias but does not list extensively all potential tools and/or steps for this purpose.

In conclusion, education and familiarization with available methodological tools, ARRIVE 2.0, PRISMA, CAMARADES, and SYRCLE (Fig. 2), is warranted to shift the research from the chancing significance to higher quality and thereby reduce research waste, unethical animal use, and ultimately unnecessary clinical studies.

### Challenges of SRs in children

Several characteristics should be considered when dealing with the pediatric population and thereby some requirements may differ from the adult population. The research in the pediatric field has been deficient.^71,72^ Many currently used treatments in children are extrapolated from adult efficacy and safety data and may result in over- or under-treatment.^71,73,74^ The rapidly developing physiology in children (neonates, infants, children, and adolescents) results in differences in pharmacology and psychology^74–77^ emphasizing the importance of the research questions to be age-specific. Evidence could be summarized and presented in recommendations for the relevant age group. However, the first problem arises from the definition of newborns, infants, children, adolescents, and adults across the studies.^78–80^ Indeed, the age of the sample population was not defined in approximately 1/3 of Cochrane reviews in children.^78^ This confusion in the definition of “child” and other age-related terms is present among the databases, leading to possible flaws in electronic search.^80^ Moreover, there is inconsistency in reporting the age of the study population in titles.^81^ To overcome this problem, specific filters for electronic search for primary studies in the pediatric population were developed.^82,83^ Kastner et al. demonstrated that the combination of MESH terms and keywords for the MEDLINE database resulted in high sensitivity and specificity (for clinical pediatric studies – 98% and 81.2% and for neonatology – 95.3 and 83.6%, respectively).^82^

Another aspect to keep in mind in pediatric SRs is the consideration of age-related physiological changes in defining the interventions. The rationale of the specific intervention should be defined for a distinct age group specifying age-related dosage, route of administration, duration of therapy, bioavailability, and other pharmacokinetics and pharmacodynamics aspects.^77^ For example, the total body water content (%) in the full-term newborn is approximately 80%, and decreases over the first year of life to approximately 60%, reaching the adult level.^84^ Thus, the water-soluble drugs have a higher volume of distribution in neonates compared to older infants and adults. This results in the need for dose adjustment. In addition to pharmacokinetic aspects, even pharmacodynamics may be influenced by developing physiology and produce different responses on intervention. A good illustration of that is the data on selective serotonin re-uptake inhibitors (SSRI) that are used for treatments of depressive and anxiety disorders. Differently from the adult population, SSRIs increase the risk for suicidal behavior, aggression, and akathisia in children and adolescents.^85^

Not only justifying the intervention but also clarifying the comparison is crucial in pediatric SRs. Commonly used comparisons in pediatric trials are “standard care” which may happen to be an off-label drug or placebo. When the “standard care” is an off-label drug there is the underlying problem of insufficient safety and effectiveness data, which raises the ethical dilemma of protecting children from research risks against not approved therapies.^86^ When a placebo is used as a comparison one should have in mind that it may have a higher response rate in children and adolescents compared to adults.^85,87^ This may ultimately introduce the underestimation of the placebo effect and overestimation of the drug effect if the drug efficacy is extrapolated from adult trials.^88^

Likewise in adult SRs, the outcomes should be determined at the planning stage of the review. Considering the age-related maturational process, some benefits and harms of the intervention may appear later in life. Acknowledging this may influence which study designs and outcomes should be included in SR. For instance, a recent scoping review on attention deficit hyperactivity disorder (ADHD) brings up the limitations of the evidence on long-term outcomes of the intervention in children and adolescents with ADHD.^89^ It suggests the overdiagnosis and overtreatment in children and adolescents with ADHD, highlighting the gaps in evidence regarding the long-term benefits and harms of diagnosis and treatment of children with milder symptoms.^89^ Such “gaps in evidence” may be the outcome of the interest of SR even though they are not identified in the primary studies. The primary outcomes of the SR must be specified a priori to avoid the outcome reporting bias.^90^

A special consideration should be made for perinatal/neonatal medicine due to the unique characteristics of this population compared to older patients. Additionally, the outcome measures are different based on gestational age at birth and related to prematurity itself, such as intraventricular hemorrhage, chronic lung disease, retinopathy of prematurity, and necrotizing enterocolitis. Additionally, because of the immaturity of several organ systems and critical illness in the neonatal period, there are long-term consequences on development. An overview of the challenges in children and juvenile animals is presented in Table 3.

**Table 3:** Challenges of SRs in children and juvenile animals.

Challenge	Possible solutions	
	Children	Juvenile animals
Age group definition: inconsistency across primary studies	Adherence with PRISMA guidelines Specifying age in the title	Adherence with PRISMA guidelines Specifying age in the title Utilizing the animals of corresponding age to the human children for the particular disease, intervention and outcome
Electronic search: inconsistency across different databases	Specifying age in the title	Specifying age in the title
Choice of appropriate animal model	_	Recognition of age-related maturational process of the organ systems in relation to the disease, intervention and outcome Collaboration with veterinary
Choice of intervention and comparison	What is known from the preclinical studies? Is there a preclinical SR on the topic? Explain the rationale for the specific age-group Acknowledge the age-related maturational process	Explain the rationale for the selection of the model, age of the animal and its relation to the clinical settings What clinical data is available at the moment?
Definition of the short- and long-term outcomes	Consider that some benefits and harms may appear in the following years The primary outcome of SR is not necessarily the primary outcome of the primary studies Define all outcomes a priori	Define all outcomes a priori Choose the appropriate model and age to assess the outcomes that relates to the clinical settings The primary outcome of SR is not necessarily the primary outcome of the primary studies
Consider the possible influence of excipient	-	Recognition of age-related maturational process of the organ systems Consider collaboration with veterinary
Number of animals used	-	Register protocol online Simplify the design Use the sample size calculation Report the number of animals at study entry
Missing reporting of sex: sex is biological characteristic with subsequent impact on physiology Is there sex effect?	Report clearly the sex of the participants of the study	Report clearly the sex of all animals (at the entry of the study, for the outcome measurement)
Missing data on mortality	Report this data clearly	Report this data clearly – it will increase the transparency
Use one species of animals: avoid animal waste	-	One specie should be sufficient (EMA regulatory guidelines)
Heterogeneity	Varies depending on the age-group and meta-analysis	Could different injury models be combined in the meta-analysis? Could different species data be pooled in the same meta-analysis?
Guidelines for reporting SRs: good to familiarize already at review planning phase	Specific guidelines for pediatric population are missing. PRISMA-Protocol Children (PRISMA-PC) and PRISMA-Children (reporting) are under development	Specific guidelines for juvenile animals are missing. Follow PRISMA guidelines until eventually the adaption for juvenile animals will be developed
Assessment of certainty of evidence	GRADE	GRADE

Considering the above-mentioned differences in the pediatric population and to increase the quality, completeness, and transparency of pediatric SRs, the proposal for the development of an extension of PRISMA guidelines, PRISMA-Protocol Children (PRISMA-PC) and PRISMA-Children (reporting), was made.^72,80,91^ The last update on protocol status was in May 2023 and it is expected that the PRISMA-C statement will be published in Q4 2023 (https://lab.research.sickkids.ca/enrich/reporting-standards/prisma-c-prisma-pc/).

## Challenges of SRs in juvenile animals

Similarly to human children, the age of the animals is one of the major determinating factors, given the continuous development and related to changes in body composition and physiology. Recently, van der Laan and colleagues demonstrated a wide variety of the ages of animals at the start of the pharmacological compounds (a total of 15 different compounds were used).^92^ For example, a compound was given at postnatal day (PND) 28 in juvenile rats while the planned pediatric age for the study was neonatal meaning that rats at PND 28 were too old (neonatal period in rats is considered to be up to PND10).^92–94^ The authors identified that in four studies a compound was started at PND 7 while the targeted pediatric age started at six years (postnatal weeks 3-6 in rats), indicating that the drug of investigation was given way too early.^92^ One of these early started compounds for the treatment of ADHD led to the development of novel behavioral effects (increased agitation, tenseness, aggressiveness, followed by decreased activity), suggesting that this drug affects the neurodevelopmental processes during brain development, resulting in changes in neuropharmacological response and altered behavior later in life.^92^ Sometimes animals may not be juvenile but the model might mimic a pediatric condition. Examples include models of hyperoxia models of bronchopulmonary dysplasia in rodent studies.^95^ It is therefore pivotal to acknowledge the developmental stage of animals in relation to the children´s treatment period, which was violated in some of the above studies, compromising the reliability of the results. Thus, animal age should be rigorously justified when dealing with juvenile animal studies.

In pharmacological studies in juvenile animals, even the use of excipients should be considered. The formulations of the drugs, including excipients, are commonly the same as in adults. However, due to the different development stages of the organs of young animals, some of the excipients may be harmful. For example, the use of propylene glycol as an excipient resulted in the mortality of mice.^96^ This means that excipient toxicity may influence the outcomes and the reliability of the results.

Another important aspect to highlight is the number of animals used in longitudinal pharmacological juvenile studies by introducing animals at different phases of the study, sometimes using more than one animal species (even though EMA regulatory guidelines indicate that one species is sufficient), and the willingness to cover multiple outcomes (complicated design) resulting in a high number of animals.^92,94^ Differently from the human studies, the mortality rate is rarely reported as well as the sex of animals. Therefore, the sample size calculation, appropriate study design, and scientific justification of animal model choice should be designed adequately.

Once studies have been included in a SR, caution is needed to ascertain whether pooling studies in the same meta-analysis, in separate meta-analyses, or narratively. For instance, studies conducted in different species or strains might lead to substantial heterogeneity, due to different responses to the same intervention. In addition, different injury models may also cause concern about the appropriateness of combining those studies in the same analysis.

Consequently, juvenile animal SRs need to deal with the weaknesses of primary studies. They have, however, the potential to reveal and highlight these weaknesses in terms of design, conduct, and reporting, as well as the lack of understanding of age relevance and its relation to specific pediatric populations, and in some cases lack of species-specific knowledge of biology and physiology. The overview of the challenges in children and juvenile animals is presented in Table 3.

Finally, GRADE guidance is needed to assess the certainty of the evidence of animal studies following well-defined criteria. For example, the GRADE domains´ indirectness and dissemination bias present different challenges than those in clinical studies.

## Translational value of preclinical research

Several methodological, conductive, and reporting problems of animal studies have been clearly shown by critically summarizing the available data in SRs. Therefore, the translational value of animal studies has been questioned.^52,62,97–101^ In the recent scoping review by Leenaars et al. of 121 reviews and “umbrella”-studies with meta-analysis on translational value of animal studies was demonstrated that the concordance rate was between 0 and 100%.^99^

Two major categories were suggested to explain the discordance between animal and human data.^97,99^ The first one may be attributed to methodological weakness and biased reporting of animal studies. Acknowledging ARRIVE 2.0, CAMARADES and SYRCLE guidelines ideally may result in high-quality animal studies and major trustworthiness of the results. For individual animal studies adherence to PREPARE (Planning Research and Experimental Procedures on Animals: Recommendations for Excellence) guidelines (https://norecopa.no/prepare) is recommended.^54^ The meticulous planning will increase the likelihood of implementation of the 3Rs principles (replacement, reduction, refinement).^102^

The second category of translational failure is based on the differences between the species and is difficult to address.^103^ It has never been scientifically proven that animals are predictable for human outcomes.^104^ Both animals and humans have sophisticated physiology and biology resulting in unpredictability of various degrees.^105^ Since the publication of the 3Rs principles,^102^ the focus of animal research was mainly on ethical aspects and regulations rather the scientific validity. Preclinical SRs highlighted flaws in the internal and external validity of animal studies. Consequently, it has been suggested to try to replace animal studies with new experimental techniques and methods based on human biology whenever possible.^100^ These approaches include sophisticated in-vitro cell models (organoids, organs-on-a-chip), computer-based models, and artificial intelligence.^100^ The modification of original 3Rs^102^ has been proposed: replacement (over reduction and refinement), research, and relevance (to humans rather than non-human animals).^100^

Currently, the research regarding these two viewpoints continues in parallel.^99^ Despite some inevitable difficulties, it is fundamental to improve animal study design, conduct, and report. Several methodological tools are available and the education of the research is a cornerstone to change the mentality, ultimately resulting in the transparency, study quality, and availability of the raw data for the research community.

## Conclusions

SRs of preclinical studies aim to assess the benefits and harms of a specific intervention. It is imperative for SRs to adhere to methodological standards, which are freely available. These include registration of the protocol, implementation of the guidelines for assessing the risk of bias, quality of the studies, and certainty of the evidence. The findings of well-conducted SRs of preclinical studies have the potential to provide a reliable evidence synthesis to guide the design of future preclinical and clinical studies.

## Supplementary information

Supplementary material

## Data Availability

Data sharing does not apply to this article as no datasets were generated or analyzed during the current study.
