# Supplementary material for: Value of preclinical systematic reviews and meta-analyses in pediatric research
Source: Pediatr Res. 2024 Apr 13;96(3):643–53. doi: 10.1038/s41390-024-03197-1 (PMC11499280; doi:10.1038/s41390-024-03197-1)
Supplement: Supplementary file 1 — Supplementary material [file 41390_2024_3197_MOESM1_ESM.pdf]

Number of pediatric studies in humans and animals, 2023-10-17.

All searches were conducted in PubMed. No language filters or publication date limits were used.

Animal newborns

(newborn*[Title/Abstract] OR "new born"[Title/Abstract] OR "new borns"[Title/Abstract] OR "newly born"[Title/Abstract] OR neonat*[Title/Abstract] OR neonatal[Title/Abstract] OR preterm[Title/Abstract] OR "pre term"[Title/Abstract] OR preterms[Title/Abstract] OR pup[Title/Abstract] OR pups[Title/Abstract] OR baby[Title/Abstract] OR babies[Title/Abstract] OR "Animals, Newborn"[Mesh]) AND "Animals"[Mesh] 580,789

Human newborns

(newborn*[Title/Abstract] OR "new born"[Title/Abstract] OR "new borns"[Title/Abstract] OR "newly born"[Title/Abstract] OR neonat*[Title/Abstract] OR neonatal[Title/Abstract] OR preterm[Title/Abstract] OR "pre term"[Title/Abstract] OR preterms[Title/Abstract] OR baby[Title/Abstract] OR babies[Title/Abstract] OR low birth weight[tiab] OR low birthweight[tiab] OR infant, newborn[Mesh]) AND ("age groups"[Mesh] OR humans[Mesh]) 795,363

Human children, including newborns

(newborn*[Title/Abstract] OR "new born"[Title/Abstract] OR "new borns"[Title/Abstract] OR "newly born"[Title/Abstract] OR neonat*[Title/Abstract] OR neonatal[Title/Abstract] OR preterm[Title/Abstract] OR "pre term"[Title/Abstract] OR preterms[Title/Abstract] OR baby[Title/Abstract] OR babies[Title/Abstract] OR low birth weight[tiab] OR low birthweight[tiab] OR infant, newborn[Mesh] OR child[Mesh] OR infants[Mesh] OR adolescent[Mesh] OR child*[tiab] OR infant*[tiab] OR adolescen*[tiab] OR boy[tiab] OR boys[tiab] OR girl*[tiab] OR toddler*[tiab] OR teenage*[tiab] OR teen[tiab] OR preschool[tiab] OR schoolage[tiab] OR school age[tiab] OR pre school[tiab]) AND ("age groups"[Mesh] OR humans[Mesh]) 4,292,659
